# First Field Release of a Genetically Engineered, Self-Limiting Agricultural Pest Insect: Evaluating Its Potential for Future Crop Protection

**DOI:** 10.3389/fbioe.2019.00482

**Published:** 2020-01-29

**Authors:** Anthony M. Shelton, Stefan J. Long, Adam S. Walker, Michael Bolton, Hilda L. Collins, Loïc Revuelta, Lynn M. Johnson, Neil I. Morrison

**Affiliations:** ^1^Department of Entomology, AgriTech, New York State Agricultural Experiment Station, Cornell University, Geneva, NY, United States; ^2^Oxitec Ltd, Milton Park, Abingdon, United Kingdom; ^3^School of Biological Sciences, Norwich Research Park, University of East Anglia, Norwich, United Kingdom; ^4^Cornell Statistical Consulting Unit, Cornell University, Ithaca, NY, United States

**Keywords:** biotechnology, engineered, insect, transgenic, *Plutella*

## Abstract

Alternative, biologically-based approaches for pest management are sorely needed and one approach is to use genetically engineered insects. Herein we describe a series of integrated field, laboratory and modeling studies with the diamondback moth, *Plutella xylostella*, a serious global pest of crucifers. A “self-limiting” strain of *Plutella xylostella* (OX4319L), genetically engineered to allow the production of male-only cohorts of moths for field releases, was developed as a novel approach to protect crucifer crops. Wild-type females that mate with these self-limiting males will not produce viable female progeny. Our previous greenhouse studies demonstrated that releases of OX4319L males lead to suppression of the target pest population and dilution of insecticide-resistance genes. We report results of the first open-field release of a non-irradiated, genetically engineered self-limiting strain of an agricultural pest insect. In a series of mark-release-recapture field studies with co-releases of adult OX4319L males and wild-type counterparts, the dispersal, persistence and field survival of each strain were measured in a 2.83 ha cabbage field. In most cases, no differences were detected in these parameters. Overall, 97.8% of the wild-type males and 95.4% of the OX4319L males recaptured dispersed <35 m from the release point. The predicted persistence did not differ between strains regardless of release rate. With 95% confidence, 75% of OX4319L males released at a rate of 1,500 could be expected to live between 3.5 and 5.4 days and 95% of these males could be expected to be detected within 25.8–34.9 m from the release point. Moth strain had no effect on field survival but release rate did. Collectively, these results suggest similar field behavior of OX4319L males compared to its wild-type counterpart. Laboratory studies revealed no differences in mating competitiveness or intrinsic growth rates between the strains and small differences in longevity. Using results from these studies, mathematical models were developed that indicate release of OX4319L males should offer efficacious pest management of *P. xylostella*. Further field studies are recommended to demonstrate the potential for this self-limiting *P. xylostella* to provide pest suppression and resistance management benefits, as was previously demonstrated in greenhouse studies.

## Introduction

Arthropod pests cause an estimated >$470 billion in lost agricultural crops worldwide (Culliney, 2014). The main tool for controlling such pests is the use of insecticides, the global annual market value of which is projected to reach $16.44 billion by 2019 (Statistica, 2019).

Insecticides will remain an important component of integrated pest management (IPM) programs but there are concerns about their off-target effects. Furthermore, resistance to insecticides is a growing problem, with 586 insect species known to be resistant to one or more insecticides (Sparks and Nauen, 2015). Other tactics will increasingly play a role in pest management in the future. Already the use of genetically engineered, insect-resistant crops (i.e., Bt crops expressing insecticidal proteins from the bacterium *Bacillus thuringiensis*) over the last two decades has played a major role in reducing the use of traditional insecticides in cotton, maize and other crops (James, 2017). As with traditional insecticides, however, the efficacy of Bt crops is threatened by the emergence of insects resistant to the Bt proteins expressed in them (Tabashnik and Carrière, 2017).

Genetic pest management goes beyond using genetically engineered pest-resistant crops and now includes genetic control of the pest itself. A predecessor of such methods is the sterile insect technique (SIT), in which sterile insects are released into wild populations of the same pest as a management intervention. This concept was independently conceived in the 1930s and 1940s by geneticist A. S. Serebrowskii in Moscow; tsetse field researcher F. L. Van der Planck in what is now Tanzania; and E. F. Knipling at the U.S. Department of Agriculture (USDA) (Klassen and Curtis, 2005). Van der Planck and Serebrowskii focused on sterility resulting from hybrid crosses between different species or different genetic strains. Knipling pursued the use of ionizing radiation to induce dominant lethal mutations causing the effect of sterility in treated insects (Knipling, 1955).

An early and on-going success has been the use of the radiation-based SIT against the New World screwworm, *Cochliomyia hominivorax*, a pest of livestock in the Americas (Gould, 2008). Decades-long international campaigns have suppressed and eradicated the New World screwworm from the USA and much of Central America and the Caribbean, with significant economic benefits (Vargas-Terán et al., 2005). A number of other pest insects have been successfully targeted by the SIT with associated reduction in the necessity for chemical control means; however, there are drawbacks to radiation-based SIT programs. The major limitation of many current SIT programs is that, in the absence of efficient sex-sorting methods, males and females are both released, which with many pests is likely to increase crop damage and/or reduce per-male efficiency (Rendón et al., 2004). Radiation can also have a negative impact on the performance of sterilized males in the field, reducing its economic feasibility (Bakri et al., 2005; Helinski et al., 2009). For many pest species, these factors prohibit use of the SIT.

Genetic approaches have been developed that overcome many of the limitations associated with SIT. One such strategy is the male-selecting, self-limiting genetic system that facilitates the mass-release of male-only cohorts of a given pest and avoids the use of potentially damaging radiation on the insects (Fu et al., 2007; Jin et al., 2013; Leftwich et al., 2014). In this system, colonies of a genetically engineered insect carry a transgene that confers female-specific mortality in the juvenile life stages, providing a means of mass-producing males which, after release into the field, find and mate with pest females. As carriers of the male-selecting, self-limiting gene, the female progeny of these released males cannot survive: with sustained releases of self-limiting males, females in the next generation are reduced, leading to population suppression. Provision of tetracycline (or suitable analogs) in the diet of larvae represses the engineered female mortality gene, allowing colonies of these insects to reproduce as normal to enable mass-production for large-scale application. Conversely, male carriers of this self-limiting gene survive as normal, even in the absence of tetracycline. Thus, after release of self-limiting males into the field, background, wild-type genetics from the mass-produced colony are introgressed into the target (wild) pest population via surviving male offspring. If the self-limiting colony comprises insects susceptible to Bt proteins (or to insecticides in general), studies indicate that sustained releases of self-limiting males can delay or even reverse the resistance developed in the target population to Bt proteins produced in genetically engineered crops (Alphey et al., 2007, 2009; Harvey-Samuel et al., 2015).

Diamondback moth, *Plutella xylostella*, is a global pest of crucifer crops estimated to cause losses of $4–5 billion annually (Zalucki et al., 2012). This species is a particularly damaging pest because of its high reproduction rate and its ability to develop resistance to most insecticides, including diamides and Bt proteins (Shelton et al., 1993; Talekar and Shelton, 1993; Zhao et al., 2006; Wang and Wu, 2012). In previous greenhouse studies with a self-limiting strain—called “OX4319L”—of *P. xylostella*, sustained introductions of self-limiting males into wild-type populations led to rapid population decline, then elimination (Harvey-Samuel et al., 2015). In the same greenhouse experiments using broccoli plants, relatively low-level releases of OX4319L males in combination with broccoli plants expressing Cry1Ac (Bt broccoli) suppressed pest population growth and delayed resistance to Bt in the *P. xylostella* population (Harvey-Samuel et al., 2015). With the increasing threat of insect resistance to Bt crops, the application of self-limiting insects to delay or reverse the development of resistance, while providing pest control, demonstrates the compatibility of using these two types of genetic pest control (Alphey et al., 2007, 2009; Harvey-Samuel et al., 2015).

The promising results achieved with self-limiting *P. xylostella* suggest that further trials are justified. Herein we report results of open-field releases, with supplemental laboratory studies, assessing the performance of self-limiting *P. xylostella* and its potential as a biological control agent. Performance measures were selected as relevant to future operational deployment: field dispersal and persistence determining spatial and temporal release strategies and mating competitiveness and longevity. Good performance in these metrics will influence male mating effectiveness in the field and, therefore, efficacy of this vertically transmitted pest control strategy. Results from these field and laboratory studies were used to develop a mathematical model describing how releases of OX4319L males could reduce or prevent outbreaks of *P. xylostella* under field conditions.

The studies described here, conducted in New York State, represent the first open-field experiments with a self-limiting strain of an agricultural insect pest. Studies were conducted under a federal permit and state and university requirements. Data from the open-field releases provide empirical evidence of the persistence, survival, and distance traveled of OX4319L moths, compared to a wild-type strain, under conditions of the trials. These data will be useful from a management perspective, and for further testing or commercial use of this, or similar, strains of self-limiting insects.

Previous studies have been conducted in Arizona using a radiation-sterilized genetically engineered pink bollworm strain that, rather than carrying a self-limiting trait, carried a genetically-engineered fluorescent protein marker, as an addition to the SIT program against this agricultural pest (Simmons et al., 2011). Those studies were followed by multiple successful trials with a genetically engineered, self-limiting strain of the mosquito, *Aedes aegypti*—the primary vector of dengue, Zika, chikungunya, and yellow fever—in the Cayman Islands, Brazil, Panama and Malaysia (Harris et al., 2011, 2012; Lacroix et al., 2012; Carvalho et al., 2015; Gorman et al., 2016).

## Materials and Methods

Several sets of complementary studies, designed to compare biological parameters between the self-limiting strain of *P. xylostella*, OX4319L, and a wild-type strain, were conducted in open-field releases and in the laboratory. In each of the experiments, we compared aspects of the insect colonies described below. All experiments were performed at Cornell University's New York State Agricultural Experiment Station (NYSAES) in Geneva, NY during 2017–8, with field releases of OX4319L conducted in September 2017. Experiments were conducted under the USDA Animal and Plant Health Inspection Service, Biotechnology Regulatory Service permit 16-076-101r.

### Insect Colonies

Two strains of *P. xylostella* were utilized for the tests and both were reared in separate walk-in environmental chambers set at 25°C on a 16:8 light to dark cycle. The OX4319L self-limiting strain of *P. xylostella* shows tetracycline-repressible, female-specific mortality: in the absence of tetracycline or suitable analogs in the larval diet, females die as larvae or pupae, whereas males survive as normal. On larval diet containing adequate concentrations of tetracycline, both sexes survive to adulthood. OX4319L insects also carry a DsRed2 fluorescent protein marker, which is visible under a fluorescence microscope in all life stages other than eggs, allowing personnel to visibly distinguish OX4319L insects from wild-type counterparts. The presence of the transgene can also be verified by PCR. The GA strain was captured from Omega Co., Georgia, USA in March 2014, and thereafter has been maintained in a laboratory on artificial diet for the larval stages. For the generation of male insects that were released, both strains were reared on diet that did not contain tetracycline (for male-only production of OX4319L) but did contain streptomycin.

### Field Studies

#### Field Site

A field on the research farm managed by NYSAES was prepared according to standard practices. The chosen field was secluded from other crucifers on the farm and surrounded by woods on three sides. On 22–23 June 2017, cabbage (cv “Cabton”) was transplanted into a field with the longest rows in the middle of the field and progressively shorter rows moving outward to create a circular field of 2.83 ha, with a diameter of 190 m. A 10 m buffer of bare ground was maintained around the perimeter of the circle. Plants were grown under standard practices until the release of the trial insects.

#### Field Release and Monitoring

Males of each strain, <24 h post-eclosion, were used for all releases. Prior to release, the sex of the moths was determined in the laboratory by examining adult genitalia while moths were anesthetized with CO_2_. Moths were allowed to fully recover before being briefly anesthetized again to coat them with fluorescent powder (Day-Glo Corp., Cleveland, OH) and transferred to a 6-L plastic release container with lid (Berry Corp., Evansville, NC), in which they were held for 3–4 h before being released in the field.

#### Field Releases

Moths were released in the center of the 2.83 ha cabbage field by opening the container and allowing them to fly. Insects that did not immediately fly were placed on a 0.8 m high table and given more time to fly away. We conducted releases with different numbers of male moths to investigate whether the number of moths released from a given point may significantly affect their behavior. A total of six releases were made. One release of 1,000 moths of each strain was made in the evening of 8 September and two releases of 2,500 moths of each strain were made during the evenings of 12 September and 14 September. Both strains on each release date were coated with the same fluorescent-colored powder to determine release date of recaptured moths; strain identification for each colored moth was verified by PCR. A single release of 1,500 and 1,000 moths, in which each strain was dusted with a different color, was made the evenings on 26 September and 27 September, respectively. On 28 September, 1,500 OX4319L and 1,400 GA males (the lower rate reflected available number of GA insects) coated with the same color were released. Strain identification for this release was also determined by PCR.

#### Recapturing Insects

Prior to releases, 48 traps were placed in the field in concentric circles at the following distances from the release site in the center of the field: 2 traps at 7 m, 4 traps at 14 m, 8 traps at 21 m, 10 traps at 28 m, 12 traps at 35 m, 4 traps at 55 m, 4 traps at 75 m, and 4 traps at 95 m. Traps in a given concentric circle were equidistant from each other. This design was developed based on a previous release of wild-type *P. xylostella* (the “Vero Beach” strain), in which ca 80% of moths that were recaptured in pheromone traps were captured 7–35 m from their release site (Bolton et al., 2019). Traps consisted of an inverted 355-ml Styrofoam cup, with a 3.3 cm-wide plastic rim at the base coated with Tanglefoot^®^ (Olson Products Inc., Medina, OH), and were secured to fiberglass poles ~0.5 m from the ground (just above the plant canopy) with a pheromone lure (Diamondback lure, Alpha Scents Inc., West Linn, OR) attached ~2 cm above the trap. The trap design and layout were similar to previous studies conducted to monitor *P. xylostella* moths (Musser et al., 2005, Bolton et al., 2019). Each trap was collected and replaced daily after each release if any *P. xylostella* moths were present on it, until no marked *P. xylostella* were detected on any traps for 2 consecutive days. Due to rain that made the field inaccessible, no traps were collected on 29 September and 9 October, which corresponds to the days post-release for the following releases: days 3 and 13 for Release 4, days 2 and 12 for Release 5, and days 1 and 11 for Release 6, respectively. The fluorescent powder color (determined by visual inspection under UV light), trap location, and collection date were recorded for each recaptured moth. Individual moths were stored in 100% ethanol at 20°C for later PCR analysis.

#### Sample Identification

For the releases in which both strains were marked with the same fluorescent powder, PCR genotyping was used to identify the strain of each recaptured moth. For trap samples with more than 20 moths of a given color, 20 moths were randomly selected for PCR genotyping. Insect samples underwent PCR genotyping using the following conditions to verify that they were either OX4319L or wild-type.

To purify sample genomic DNA for PCR genotyping, we used two methods: either using a Purelink Genomic DNA kit (Invitrogen, Carlsbad, USA); or placing each sample in a Nunc Immuno Plate with 50 μL 300 mM sucrose solution (5.15 g sucrose, 0.875 g NaCl and 3 mL 1M Tris-HCl pH 8.0 in 50 mL ultrapure water), homogenizing the sample, incubating the sealed plate at 95–99°C for 9 min, spinning the samples at 4,000 rpm for 2 min, and placing samples on ice for 5 min before transferring the supernatant to a new 96-well plate. Each sample was genotyped by PCR [2 min at 95°C, 35 × (15 s at 95°C, 15 s at 62°C, 15 s at 72°C), and 5 min at 72°C] to detect two sequences, analogous to those described by Walters et al. (2012): one spanning the 5′ junction of the OX4319L genomic insertion (primers: “OX4319L-Pxy F2,” sequence available on request; with “PB5-out,” 5′-CTCTGGACGTCATCTTCACTTACGTG-3′); and the other spanning the wild-type locus of the same OX4319L genomic insertion site (primers: “OX4319L-Pxy F1”; with “OX4319L-Pxy R1,” sequences available on request). Further PCR genotyping was undertaken on some samples, where the result of the first genotyping run was uncertain [2 min at 95°C, 40 × (15 s at 95°C, 15 s at 62°C, 15 s at 72°C), and 5 min at 72°C].

The identity of the remaining moths for that trap was estimated based on the proportion of each strain determined by PCR genotyping. For all cases where PCR genotyping failed, for instance due to inadequate genomic DNA template from a fragmented insect sample, the proportion of each strain was determined by the remaining samples associated with the same trap that were successfully genotyped. Trap collections from which none of the moths could be genotyped, either directly by PCR or indirectly by estimation (as described above), were not included in further analysis.

For each release (marked by a given powder color), the total of all the moths recaptured of each strain at each trap distance was determined for each day released (marked) moths were detected in the field. Each release had very high recapture rates at 7 m, indicating that released moths were over-sampled in traps at this distance; thus, all counts from 7 m were excluded from dispersal analysis but not from persistence analysis.

#### Persistence of Field-Released *P. xylostella*

To determine the persistence (i.e., how long released moths could be trapped in the field) of each strain in the field during each release, the relative cumulative proportion of moths recaptured (rc#R_p_) each day for each strain within a release was calculated as follows: the cumulative number of moths of each strain recaptured daily (at all trap distances including 7 m) was divided by the total caught during that release to yield the cumulative proportion caught. This proportion was subtracted from the total number caught for that release to yield the relative cumulative proportion. To satisfy model assumptions, a log(× + 0.001) transformation of rc#R_p_ was used for statistical analysis of persistence.

#### Survival of Field-Released *P. xylostella*

The daily sum of all moths recaptured at all trap distances (including 7 m) for each strain was calculated. This daily sum was divided by the number of moths initially released less those previously recaptured to yield the proportion surviving each day. The relative proportion surviving (rpS) is the daily proportion surviving divided by the proportion recaptured the first day post-release. Because some early daily observations were not undertaken due to rain (days 1 and 3 for one of the 1,500-moth releases and day 2 for one of the 1,000-moth releases) and the proportion recaptured after the first day post-release (the first day that moths were recaptured after release) was greater than the proportion recaptured on the first day post-release resulting in the relative proportions surviving >1, only the data from the two 2,500-moth releases were used for analysis.

#### Mean Distance Traveled Calculation

The mean distance traveled (MDT) for each release was calculated according to the method of Morris et al. (1991) for each strain within each release rate using the number of moths recaptured at each trap distance > 7 m for each strain. The relative area for each annulus associated with each trap distance was calculated as the difference of the areas of a given (trap distance) anulus from the previous one divided by the area of the 14 m circle. The annulus distance used here was the distance of the trap from the release point at a given distance. For each release and day post-release, the cumulative number of each strain caught at each trap distance was determined by adding the number caught on the previous day to the current day for the entire monitoring period. The value from the last day monitored for a given release rate and strain was continued (unchanged) for the days up to the longest monitored period (14 days). These values were used to calculate the cumulative total estimated recapture (ER) and the product of the trap distance and cumulative total estimated recapture (ERXD) for each day post-release. To determine the daily MDT for each release and strain, the cumulative ERXD was divided by the cumulative ER for each day post-release. The overall daily MDT is the average of the daily MDT for each release. The MDT from the last day post-release is also the overall release rate for each release rate and strain. To satisfy model assumptions, $\sqrt{MDT}$ was used for statistical analysis.

#### Dispersal of Field-Released *P. xylostella*

To determine overall dispersal from the release point, the relative cumulative proportion of moths recaptured (rc#R_d_) at each trap distance beyond 7 m for each strain within release was calculated as follows: the cumulative number of moths of each strain recaptured at each trap distance was divided by the total caught during that release (excluding those caught at 7 m) to yield the cumulative proportion caught at each trap distance. This proportion was subtracted from the total number caught for that release to yield the relative cumulative proportion. To satisfy model assumptions, a log(× + 0.001) transformation of rc#R_d_ was used for statistical analysis.

A separate analysis was conducted to determine the effect of strain and release rate on the proportion of moths recaptured relative to the number of moths released. For each release, the number recaptured at each trap distance for each strain was divided by the number of moths released for that strain (pR). To satisfy model assumptions, $\sqrt{\left(pR\right)}$ was used for statistical analysis.

### Laboratory Studies

#### Mating Competition

The ability of OX4319L males to mate with GA females in competition with GA males was assessed using two methods: by determining the paternity of larvae of individual females throughout their lives, and by determining the paternity of larvae collected from a group of 20 GA females every 48 h up to 7 days. Only OX4319L males can pass on the fluorescent marker to their offspring, therefore the paternity of any larvae that showed DsRed2 fluorescence was assigned to OX4319L.

#### Competitive Mating With Individual Females

Two <24 h-old virgin GA females were placed with two <24 h-old GA males and two <24 h-old OX4319L males into each of six cages (60 × 60 × 47 cm) for 24 h. After this period, females were isolated individually to a 10 × 100 mm Petri dish containing an 18 × 18 mm coverslip treated with cabbage juice to induce oviposition. Thirty-one females were transferred every 48 h to a new dish with a freshly treated coverslip three more times (up to 7 days post-mating). To catch first-instars, each Petri dish was ringed on the inside with electrical tape (sticky side facing inward) to capture wandering larvae and covered. The number of eggs laid and the number of resulting larvae and fluorescent larvae were counted at 10 × magnification using an Olympus SZX16 stereo microscope with a 100-W high pressure mercury burner (model #BH2-RFL-T3) and the combination of an Olympus SZX RFL3 filter with a barrier filter B580IF (to emit 520–550 nm light) within a week of exposure and recorded. The proportion of fluorescent larvae was recorded.

#### Competitive Mating With a Group of Females

Twenty 48 h-old virgin OX4319L and twenty 48 h-old virgin GA males were released into each of five 60 × 60 × 47 cm cages. After 4 h, twenty 48 h-old virgin GA females were released into the same cage. A 5 × 10 cm Parafilm sheet coated with cabbage juice was hung in the cage as an oviposition surface. This set up was replicated twice yielding 10 groups of females tested. Every 48 h the sheet with any *P. xylostella eggs* was collected and replaced, three more times. To catch first-instars, each sheet was placed in a 10 × 100 mm Petri dish ringed on the inside with electrical tape, as previously described, and covered. The dish was labeled with cage number, the date that the Parafilm sheet was exposed to the caged moths, and the days after the moths were released into the cages (day 1, 3, 5, and 7 post-mating). The number of eggs laid and hatched on the Parafilm sheet and total number of first-instars and DsRed2-positive larvae on the tape were counted at 10 × magnification using the microscope described above. Counts to detect DsRed2 fluorescence were conducted within a week of exposure. The proportion of DsRed2-positive larvae was used for statistical analysis.

#### Intrinsic Growth Rate

To determine if mating with OX4319L males had any effect on the reproductive output of the females with whom they mated, the following experiments were performed. Twenty-eight <24 h-old virgin GA females were released into a cage with more than 500 GA males and 30 <24 h-old virgin GA females were released into a cage with more than 500 OX4319L males, respectively, to mate for 2 h. Once mating was observed, females were placed individually in a 10 × 100 mm Petri dish with an 18 × 18 mm coverslip coated with cabbage juice. Females were transferred daily to a new similarly prepared Petri dish until death. Each dish was ringed with black electrical tape, as previously described, after the female was removed and covered. The numbers of eggs laid and first-instars caught on the tape were recorded. The intrinsic growth rate (measured as larval output, not female progeny output) for each group was calculated as the sum of the products of the daily total number of larvae (m_i_) and the proportion of surviving females (l_i_) for each group according to the method of Wilson and Bossert (1971). The daily cumulative larval output (∑l_i_^*^m_i_) was calculated by adding the previous daily larval output to the current day's larval output. To satisfy model assumptions, the square root of the cumulative daily larval output was used for statistical analysis.

#### GA and OX4319L Male Longevity

Two hundred <24 h-old virgin males from each strain were collected from three different cohorts of pupae and divided equally between two treatments with one provided a 7.5% (w/v) sucrose solution with 67 mg/L methyl-4-hydroxybenzoate daily via a soaked cotton ball; the others were not. Each moth was individually contained in a 29.6 mL plastic cup with lid and checked daily until death. For each treatment, the proportion alive was calculated and used for statistical analysis.

The treatments for male and female longevity were arbitrarily assigned a number for analysis (Treatment 1, GA or OX4319L males not provided 7.5% sucrose solution; Treatment 2, GA female mated with GA or OX4319L male; Treatment 3, GA or OX4319L male provided 7.5% sucrose solution).

### Statistical Analysis

JPM Pro 13.1.0 software (SAS Institute, Cary, NC; 2016) was used for all statistical analysis. Strain, release rate, release number, cage, replicate, female number and trap distance (for dispersal analysis relative to the number of moths released) were included in all analyses as categorical (nominal) variables. Days post-release and trap distance (for dispersal analysis relative to the number of moths recaptured used to estimate distances where 100 or 90% of moths were recaptured), days post-mating, day, and all response variables were analyzed as continuous variables. For all linear models and linear mixed effects models described below, a residual analysis plot (residual values vs. expected values) was visually examined to verify model assumptions of normality and homoscedasticity were met. Response variables were transformed as necessary to ensure that model assumptions were met. *Post-hoc* comparisons were made using Tukey's HSD method to control for multiple comparisons where *p* < 0.05 were considered statistically significant.

The overall percentage recaptured was analyzed using a linear mixed effects model with the proportion recaptured as the response variable and strain, release rate and their interaction as fixed effects and release number as a random effect.

Mean distance traveled was analyzed using a linear mixed effects model with $\sqrt{MDT}$ as the response variable and strain, release rate, days post-release and their interactions (full factorial) as fixed effects and release number as a random effect.

Dispersal data were analyzed using a linear mixed model with log(rc#R_d_ + 0.001) and $\sqrt{\left(pR\right)}$ as response variables and strain, release rate, trap distance and their interactions (full factorial) as fixed effects and release number as a random effect.

Persistence data were analyzed using a linear mixed model with the log(rc#R_p_ + 0.001) as the response variable and strain, release rate, days post-release and their interactions (full factorial) as fixed effects and release number as a random effect.

Field survival data were transformed using a Box Cox transformation [ln(rpS + 0.01)] to linearize the data and satisfy model assumptions. These transformed data were analyzed with a linear mixed effects model using ln(rpS + 0.01) as the response variable, strain and days post-release and their interaction as the fixed effects and release number as a random effect.

For both the individual and group mating competition experiments, the proportion of fluorescent larvae (p fl L) were analyzed using a linear mixed effects model with individual female number, and group number as random effects for individual and group experiments, respectively.

Data from the intrinsic growth experiment were analyzed using a linear model with the square root of (${\text{l}}_{\text{i}}^{*}$m_i_) as the response variable and mate, day and their interaction as fixed effects.

Longevity data were analyzed using a linear model with proportion alive as the response variable and strain, treatment, day and their interactions as fixed effects.

### Population Modeling Studies

A deterministic model was developed to predict the effects of different release rates of OX4319L males on target populations in future suppression programs. The time horizon was 84 days (12 weeks) simulating about three generations during one cropping season given that egg-to-adult development time is 21 days in the model. Using findings from the mark-release-recapture studies described in this paper, we assumed equal daily survival rates (0.7/day) for adults of all genotypes. The number of adults, A, of sex s and genotype g on day t is

$$\begin{array}{l} A\left(s,g,t\right)=I\left(s,g,t\right)+R\left(s,g,t\right)+0.7A\left(s,g,t-1\right)\\ +0.225E\left(s,g,t-21\right) \end{array}$$

where I is the immigration of adults, R is the number of released adults (only OX4319L males), and E is the number of adults maturing from eggs laid 21 days before. Note that A(s,g,0) = 0. In some simulations, a single immigration of wild-type *P. xylostella* occurs on the first day. In others, immigration of wild moths occurs once per week over the 84 days. Simulated releases of OX4319L males occurred either on only the first day, weekly, or every 2 weeks, with patterns sometimes matching the pattern of immigration.

Mating between males and females was assumed to be random, independent of moth genotype. Older females can mate more than once in the model. We assumed that female immigrants mate after arrival.

The dominant lethal allele kills all females except those homozygous for the wild-type. Because immigrants are homozygous for the wild-type allele, w, the only females that mate in the model are homozygous, and only released males, R, can be homozygous for the lethal allele. We account for this female mortality when calculating E(f,g,t), where f and m are designations for females and males. Fecundity was assumed to be 10 eggs/day and sex ratio of eggs was 0.5. Note that reproductive rate and mortality vary considerably in temperate North America (Harcourt, 1985; Dancau, 2018). Therefore, the values of E at time t for the viable sexes and genotypes are

$$\begin{array}{l} E\left(f,w,t\right)=10x0.5xA\left(f,w,t\right)x\;P\left(w\right)\\ E\left(m,w,t\right)=10x0.5xA\left(f,w,t\right)x\;P\left(w\right)\\ E\left(m,y,t\right)=10x0.5xA\left(f,w,t\right)x\;P\left(y\right) \end{array}$$

Where the probability that mating produces a wild-type homozygote is

$$\begin{array}{l} P(w)\;=\;\left[A(m,w,\;t)+\;0.5A(m,\;y,\;t)\right]/\left[A(m,w,\;t)+\;A(m,\;y,\;t\right)\\ \;+A(m,\;x,\;t)] \end{array}$$

And the probability that mating produces a heterozygous male is

$$\begin{array}{l} P(y)=\left[A(m,\;x,\;t)+0.5A(m,\;y,\;t)\right]/\left[A(m,w,\;t)+A(m,\;y,\;t\right)\\ \;+A(m,\;x,\;t)] \end{array}$$

For genotypes that are homozygous wild-type w, heterozygous y, and homozygous x for the OX4319L allele. Again, the E(f,y,t) and E(f,x,t) all die, and no matings can create E(m,x,t) because of lack of A(f,x,t).

## Results

The results described below describe a series of studies evaluating the behaviors of the genetically modified self-limiting strain (OX4319L) compared to a wild-type counterpart (referred to as GA). Six open-field releases were performed at three different release rates, with two co-releases of both strains for each release rate. A series of laboratory studies compared the mating competence and longevity of the two strains. Results from the field and laboratory studies were utilized to generate a predictive deterministic model for calculating the ability of different release rates of OX4319L moths to suppress wild-type populations *of P. xylostella*.

### Field Release Studies and Monitoring

#### Persistence of Field-Released *P. xylostella*

Persistence measured how long released moths were trapped in the field. Regression equations for log(rc#R_p_ + 0.001) for each strain and release rate were used to calculate the expected mean time after release (days) and related 95% confidence intervals of when 50, 75, 90, and 100% of the male moths recaptured would occur (Table 1). These estimates varied with the release rate. For example, with 95% confidence, 90% of the 1,000 OX4319L males released that would be recaptured would be expected to be recovered after 6.6–7.8 days. With 95% confidence, 90% of the 1,500 OX4319L males released, that would be recaptured, would be expected to be recaptured after 5.4–7.1 days. With 95% confidence, 90% of the 2,500 OX4319L males released, that would be recaptured, would be expected to be recaptured between 2.7 and 3.7 days, respectively. In no cases did the persistence differ between the OX4319L and GA strains.

**Table 1:** Persistence (days) of two strains of *Plutella xylostella* released in a cabbage field.

		95% CI estimates^*^ for days until			
**Strain**	**Release rate**	**50% recaptured**	**75% recaptured**	**90% recaptured**	**100% recaptured**
GA	1,000	(1.5–5.1)	(3.3–6.2)	(5.4–8.0)	(13.4–19.7)
OX4319L	1,000	(0.1–3.1)	(4.8–6.2)	(6.6–7.8)	(14.1–17.1)
GA	1,500	(0.6–1.6)	(1.6–4.1)	(3.5–5.5)	(11.0–15.1)
OX4319L	1,500	(3.5–5.1)	(3.5–5.4)	(5.4–7.1)	(12.7–17.9)
GA	2,500	(1.9–4.3)	(1.5–2.4)	(2.8–3.5)	(8.4–9.6)
OX4319L	2,500	(0.7–2.1)	(1.6–2.8)	(2.7–3.7)	(7.5–9.0)

* *Based on inverse intercept calculations using the regression equation for each strain and release rate combination with log(rc#R_p_+0.001) as the response variable and days post-release as the only fixed factor (no random factors included). See Table 2 for associated regression equations and predicted inverse intercepts*.

The difference in persistence between males from the 1,000- and those from 1,500-male release rates was not significantly different; however, the rate of persistence of both strains of males released at the 2,500-male release rate decreased significantly faster than those males released at a rate of 1,000 males (*P* = 0.011) and at a release rate of 1,500 males *(P* = 0.007) (Table 2). Persistence at the 2,500-male release rate was only 8.9 and 8.1 days for the GA and OX4319L strains, respectively, compared to >12 days for the other release rates.

**Table 2:** Regression equations and intercepts for persistence of *Plutella xylostella* strains released at three release rates.

				**Predicted inverse intercept value (days)**			
**Strain**	**Release rate**	**Regression equation**	***R*^**2**^ value**	**50% recaptured**	**75% recaptured**	**90% recaptured**	**100% recaptured**
GA	1,000	log(rc#R_p_ + 0.001) = 0.562–0.0.225^*^(days post-release)	0.64	3.7	5.0	6.8	15.7
OX4319L	1,000	log(rc#R_p_ + 0.001) = 0.763–0.0.244^*^(days post-release)	0.87	4.4	5.6	7.2	15.4
GA	1,500	log(rc#R_p_ + 0.001) = 0.181–0.0.254^*^(days post-release)	0.75	1.9	3.1	4.6	12.5
OX4319L	1,500	log(rc#R_p_ + 0.001) = 0.478–0.0.237^*^(days post-release)	0.76	3.3	4.6	6.2	14.7
GA	2,500	log(rc#R_p_ + 0.001) = 0.100–0.0.348^*^(days post-release)	0.95	1.1	2.0	3.1	8.9
OX4319L	2,500	log(rc#R_p_ + 0.001) = 0.327–0.0.408^*^(days post-release)	0.91	1.5	2.3	3.2	8.1

#### Survival of Field-Released *P. xylostella*

Another measurement of the fate of the released insects is the relative proportion surviving (rpS) and is defined as the daily proportion surviving divided by the proportion recaptured the first day post-release. Moth strain had no effect on field survival (*P*_strain_ = 0.4546), but days post-release did (*P*_days post−release_ <0.0001) (Table 3). These data fit the overall linear regression equation [ln(rpS + 0.01) = 0.828665-0.617303^*^days post-release] very well (*R*^2^ = 0.867) (Table 4). This equation could be used to estimate field survival of future releases of OX4319L under similar conditions. This estimate would be conservative because the maximum time for moths released at the 2,500-male release rate persisted in the field was the shortest (8.1 days), relative to those released at the 1,000- and 1,500-male release rates (15.4 and 14.7 days, respectively) (Table 2).

**Table 3:** Relative^*^ percent survival of two strains of *Plutella xylostella* males released on six different dates.

			**Days Post-release**													
**Strain**	**Release rate**	**Release date**	**1**	**2**	**3**	**4**	**5**	**6**	**7**	**8**	**9**	**10**	**11**	**12**	**13**	**14**
GA	2,500^†^	12-Sep-2017	100.0	40.4	38.2	21.1	1.8	1.1	1.1	0.7						
OX4319L	2,500^†^	12-Sep-2017	100.0	**111.4**^‡^	**145.5**	43.2	6.8	2.3	2.3	2.3						
GA	2,500^†^	14-Sep-2017	100.0	89.6	43.3	23.9	17.1	9.2	1.2	0.6	1.2					
OX4319L	2,500^†^	14-Sep-2017	100.0	50.7	39.0	6.0	18.1	9.1	0.0	0.0	0.0					
GA	1,000	8-Sep-2017	0.0	0.0	100.0	**140.7**	20.2	**101.3**	**101.8**	**225.2**	82.8	41.6	41.7	0.0	41.8	20.9
OX4319L	1,000	8-Sep-2017	0.0	0.0	100.0	**160.8**	81.1	**101.7**	40.9	*61.5*	20.6	*41.2*	0.0	*20.6*	0.0	0.0
GA	1,000	27-Sep-2017	100.0	—-^§^	44.5	4.5	*36.0*^¶^	*27.2*	*18.3*	*27.5*	*9.2*	0.0	0.0	0.0	0.0	
OX4319L	1,000	27-Sep-2017	100.0	—-	43.2	28.9	28.9	**275.3**	**310.2**	**437.9**	15.6	*62.4*	15.7	0.0	15.7	
GA	1,500	26-Sep-2017	100.0	15.2	—-	0.0	0.4	1.5	*3.8*	*1.9*	*1.9*	0.0	0.4	0.0		
OX4319L	1,500	26-Sep-2017	100.0	58.4	—-	8.2	1.2	*2.3*	*12.9*	*9.5*	*16.7*	*4.8*	1.2	1.2		
GA	1,400	28-Sep-2017	—-	100.0	**150.9**	**418.6**	**584.8**	**551.2**	**930.0**	44.0	29.4	29.4				
OX4319L	1,500	28-Sep-2017	—-	100.0	100.1	**1301.7**	**2929.1**	**4429.7**	**4033.8**	**327.3**	**1421.2**	**441.5**				

* *Relative to the number collected on the 1^st^ day that males of a strain were detected after a release date*.

† *Only data from 2,500 releases were used for survival analysis*.

‡ *Value in **bold** because relative % survival was >100%*.

§ *No data were recorded*.

¶ *Value in italics because relative % survival was greater than previous day*.

**Table 4:** Field survival regression estimates of *Plutella xylostella* males at 2,500 release rate.

**Term**	**Estimate**	**SE**	***p*-value**
Intercept	0.828665	0.229450	0.0010
Days post-release	−0.617303	0.042782	<0.0001

#### Distribution of *P. xylostella* Recaptured in Field Releases

In the six mark-release-recapture studies, the percent recovered varied by distance from the release site for the GA and OX4319L strains (Table 5). For the closest trapping site (7 m), there was no significant difference in the overall mean percentage recovered ± SE for the GA strain and the OX4319L strain: 51.6% ± 8.1 and 47.8% ± 4.2, respectively. The combined percentage ± SE recovered between 14 m and 35 m was 46.2% ± 7.5 for GA and 47.6% ± 4.8 for OX4319L, respectively, with no significant difference. Thus, the total proportion recaptured in the first 35 m was 97.8 and 95.4% for GA and OX4319L, respectively. Less than 5% of released moths of either strain were recovered beyond 35 m, indicating the limited dispersal of both strains. Although no significant differences between populations were observed at any specific distance for any of the releases, the overall mean percentage recaptured from the six releases was significantly higher for the GA strain.

**Table 5:** Percent recovered at different distances from the release point for two strains of *Plutella xylostella*.

Release Rate^*^	**Release Date**	**GA recovered, 7 m**	**OX4319L recovered, 7 m**	**GA recovered, 14–35 m**	**OX4319L recovered, 14–35 m**	**GA recovered, >35 m**	**OX4319L recovered, >35 m**	**Overall recaptured GA**	**Overall recaptured OX4319L**
1,000	8-Sep-2017	33.3	41.9	57.8	58.1	8.9	0.0	4.5	3.1
1,000	27-Sep-2017	30.0	43.3	70.0	44.4	0.0	12.2	6.0	9.0
1,500	26-Sep-2017	60.6	51.0	38.9	42.9	0.5	6.1	28.9	13.1
1,500^†^	28-Sep-2017	80.3	61.4	19.7	37.2	0.0	1.4	15.2	9.7
2,500	12-Sep-2017	64.1	56.0	34.1	37.3	1.7	6.7	23.2	3.0
2,500	14-Sep-2017	41.0	33.0	56.8	65.9	2.2	1.1	27.2	7.3
	Mean %^‡^± SE	51.6 ± 8.1	47.8 ± 4.2	46.2 ± 7.5	47.6 ± 4.8	2.2 ± 1.4	4.6 ± 1.9	17.5^a^ ± 4.3	7.5^b^ ± 1.6

* *Number of male P. xylostella of OX4319L and GA released*.

† *On 28-Sep-2017, 1,400 GA and 1,500 OX4319L were released*.

‡ *Mean % followed by different letters are significantly different as determined by a two-sample t-test, p = 0.0128*.

#### Mean Distance Traveled

**Figure 1:**
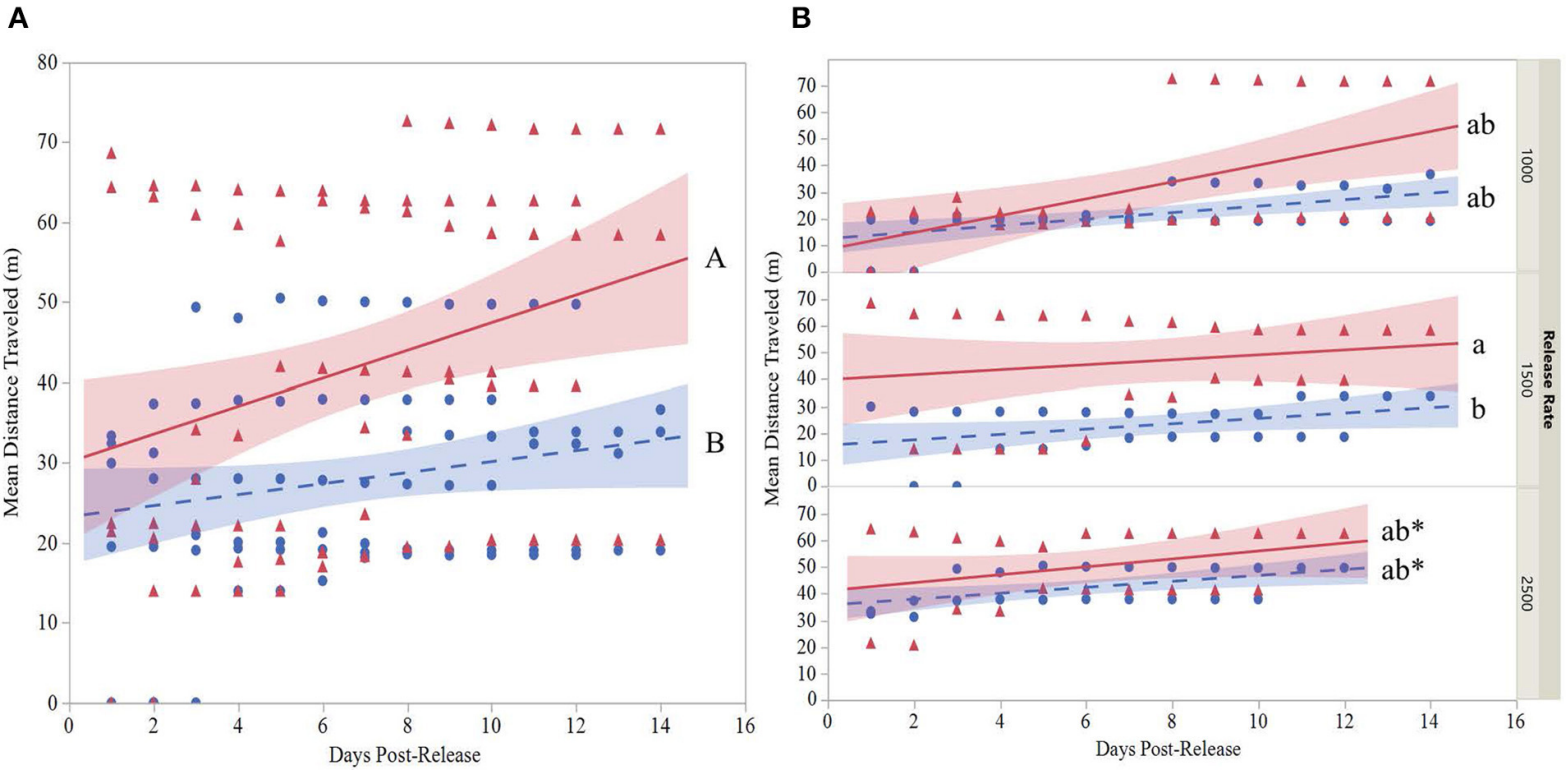
**(A)** Overall regression with 95% confidence interval bands of the mean distance traveled (MDT) for two strains (
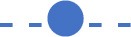
, GA; 
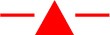
, OX4319L) of *P. xylostella* and **(B)** regression with 95% confidence interval bands at three release rates (1,000, 1,500, 2,500 males of each strain) in a 2.3 ha cabbage field (7 m traps excluded). MDT calculated according to Morris et al. (1991). Cumulative MDT analyzed with a mixed model where $\sqrt{MDT}$ is the response variable; strain, release rate and days post-release are fixed variables and release number is a random variable. *Last day observations were made at the 2,500 release rate. Overall strain lines with different capital letters (panel A) are significantly different as determined by a two-sample *t*-test, *P* < 0.001. Lines that do not share the same small letter(s) **(B)** are significantly different as determined by Tukey's HSD test, HSD_0.05,6_ = 2.89.

The mean distance traveled by strain was highly variable for each release (Table 6). Although overall, OX4319L males (50.2 m ± 15.4, mean ± SE) traveled significantly farther than GA males (29.9 m ± 5.5) 14 days post-release (two-sample *t*-test, *P* < 0.0001), this appears to be due to the 1,500 release rate because no such statistical differences were observed with the other release rates. At the 1,500-male release rate, OX4319L males traveled significantly farther than GA males at the same release rate, 49.0 m ± 9.4 and 26.2 m ± 7.7, respectively) (Tukey HSD_0.05, 6_ = 2.89). At the release rates of 1,000 and 2,500, there were no statistical differences. These results are also shown graphically in Figure 1A, which illustrates the significant differences in distance traveled over time for all releases for both strains, while Figure 1B illustrates the differences by release rates and shows significant differences between the strains only at the 1,500 release rate.

**Table 6:** Mean distance traveled [MDT] ±SE for *Plutella xylostella strains* released at three rates in field.

**Release Rate**		**1,000**		**1,500**		**2,500**		Overall^*^	
**Days Post-**		**Strain**		**Strain**		**Strain**		**Strain**	
**Release**	***N***	**GA**	**OX4319L**	**GA**	**OX4319L**	**GA**	**OX4319L**	**GA**	**OX**
1	12	9.8 ± 9.8	11.2 ± 11.2	30.0^†^	68.7^†^	32.9 ± 0.5	42.9 ± 21.5	23.1 ± 6.3	35.4 ± 13.3
2	12	9.8 ± 9.8	11.2 ± 11.2	14.0 ± 14.0	39.3 ± 25.3	34.3 ± 3.1	41.9 ± 1.3	19.4 ± 6.6	30.8 ± 11.0
3	12	20.0 ± 1.0	25.1 ± 2.9	14.0 ± 14.0	39.3 ± 25.3	43.4 ± 6.0	47.6 ± 13.4	25.8 ± 6.9	37.3 ± 8.5
4	12	19.7 ± 0.4	19.9 ± 2.3	21.0 ± 7.0	39.0 ± 25.0	42.9 ± 5.1	46.6 ± 13.2	27.9 ± 5.3	35.2 ± 8.9
5	12	19.6 ± 0.5	20.1 ± 2.1	21.0 ± 7.0	39.0 ± 25.0	44.1 ± 6.4	49.8 ± 7.8	28.3 ± 5.6	36.3 ± 8.7
6	12	20.2 ± 1.1	18.8 ± 0.1	21.6 ± 6.3	40.5 ± 23.4	44.1 ± 6.1	52.2 ± 10.5	28.6 ± 5.4	37.2 ± 9.1
7	12	19.4 ± 0.6	21.0 ± 2.6	22.8 ± 4.7	48.1 ± 13.7	44.0 ± 6.1	52.1 ± 10.6	28.7 ± 5.3	40.4 ± 7.7
8	12	26.6 ± 7.4	46.1 ± 26.6	23.0 ± 4.4	47.4 ± 13.9	43.9 ± 6.0	52.0 ± 10.7	31.2 ± 4.9	48.5 ± 8.3
9	12	26.3 ± 7.2	46.0 ± 26.4	22.8 ± 4.4	50.0 ± 9.5	43.8 ± 5.9	52.0 ± 10.7	31.0 ± 4.9	49.3 ± 7.8
10	12	26.2 ± 7.1	46.3 ± 25.9	22.9 ± 4.3	49.1 ± 9.5	43.8 ± 5.9	52.0 ± 10.7	31.0 ± 4.9	49.1 ± 7.7
11	10	25.7 ± 6.7	46.0 ± 25.7	26.2 ± 7.7	49.1 ± 9.5	49.8^†^	62.7^†^	30.7 ± 5.7	50.6 ± 9.2
12	10	25.7 ± 6.7	46.0 ± 25.7	26.2 ± 7.7	49.0 ± 9.4	49.8^†^	62.7^†^	30.7 ± 5.7	50.6 ± 9.2
13	6	25.1 ± 6.1	46.0 ± 25.7	33.9^†^	58.4^†^			28.1 ± 4.6	50.2 ± 15.4
14	6	27.9 ± 8.8	46.0 ± 25.7	33.9^†^	58.4^†^			29.9 ± 5.5	50.2 ± 15.4
Final MDT^‡^		27.9^ab^± 8.8	46.0^ab^±25.7	26.2^a^ ± 7.7	49.0^b^ ± 9.4	43.8^ab^± 5.9	52.0^ab^ ± 10.7	29.9^A^ ± 5.5	50.2^B^ ±15.4

*Traps at 7 m are not included and no observations were made after last value in column. Mixed model where$\sqrt{MDT}$ is the response variable; strain, release rate and days post-release are fixed variables and release number is a random variable. N = # releases × #strains × #release rates*.

* *Overall Strain means followed by different capital letters in last two columns are significantly different as determined by a two-sample t-test, p <0.0001*.

† *SE could not be calculated because data from only a single release was available*.

‡ *Final MDT values are from the last day post-release where observations from both replicates were made. Means followed by different small letters in bottom row (HSD_0.05, 6_ = 2.89) are significantly different*.

#### Dispersal of Field-Released *P. xylostella*

For dispersal relative to the total number of moths recaptured, there was no significant effect of strain (*P*_strain_ = 0.310), release rate (p_release rate_ = 0.685) or the interaction between release rate and strain ($P_{\text{release rat}{\text{e}}^{*}strain}$ = 0.824) on moth dispersal. Trap distance was the only factor that significantly affected moth dispersal (*P*_trap_ _distance_ <0.001). The overall regression equation log(rc#R_d_ + 0.001) = 0.0099749–0.0327758^*^(trap distance) accounted for 71% of the variation in the data (*R*^2^ = 0.71) (Table 7). This equation was used to calculate the expected means, and related 95% confidence intervals, of the distances for each strain within a given release where 90 and 100% of the male moths recaptured would occur. With 95% confidence, 90 and 100% of what would be recaptured would be expected to be recovered between 25.8–34.9 m and 85.1–100.5 m from the release point, respectively. The high degree of confidence that 100% of the moths would be recaptured within 100.5 m of their release indicates that the 2.83 ha cabbage field was of appropriate size for the dispersal study.

**Table 7:** Regression equation and predicted intercepts values for dispersal of *Plutella xylostella* released in cabbage field.

		**Expected mean distance (m)**		**95% Confidence Interval (m)**	
**Regression equation**	***R*^**2**^ value**	**90% recaptured**	**100% recaptured**	**90% recaptured**	**100% recaptured**
log(rc#R_d_ + 0.001) = 0.0099775–0.0.032776*(trap distance)	0.71	30.7	91.8	(25.8–34.9)	(85.1–100.5)

For dispersal relative to the total number of moths released, significant differences between strains (*P*_*strain*_ = 0.005), the strain by release rate interaction ($P_{\text{strai}{\text{n}}^{*}rel\;rate}<$ 0.001), trap distance (*P*_trap dist_ <0.001) and the strain by trap distance interaction ($P_{\text{strai}{\text{n}}^{*}trap\;dist}$ = 0.0068) were found (Table 8). At all distances except 14 m, there were no significant differences in the mean proportion recaptured. Few moths (≤ 0.1%) were recaptured at 55, 75 and 95 m. Overall distances, the recapture proportion was higher for GA (7.6%) than for OX4319L (3.7%).

**Table 8:** Mean percentage ± SE of *Plutella xylostella* strains recaptured at distances from release point.

	**Trap distance (m)**							
Strain^*^	**14**	**21**	**28**	**35**	**55**	**75**	**95**	**Overall Total^†^**
GA	3.3^a^ ± 0.9	2.0^ab^ ± 0.6	1.3^bc^ ± 0.4	0.8^bc^ ± 0.3	0.1^de^ ± 0.1	0.0^e^ ± 0.0	0.1^de^ ± 0.0	7.6^A^ ± 0.2
OX4319L	1.3^bc^ ± 0.3	1.0^bc^ ± 0.2	0.8^bc^ ± 0.1	0.4^cd^ ± 0.0	0.0^e^ ± 0.0	0.1^de^ ± 0.0	0.1^de^ ± 0.0	3.7^B^ ± 0.1

* *Strain means across trap distances for both strains followed by a different small letter are significantly different (HSD_0.5, 14_ = 3.57)*.

† *Overall strain totals followed by different capital letters in last column are significantly different as determined by a two-sample t-test, p = 0.0005*.

### Laboratory Studies

#### Mating Competition With Individual Females and With a Group of Females

In both scenarios, OX4319L showed similar mating performance to GA males. In both studies, all females produced a mixture of DsRed2-positive and DsRed2-negative larvae. Overall the mean (95% confidence interval) for the proportion of DsRed2-positive larvae (i.e., offspring of OX4319L males) was 52.0% (39.1–64.9%) and 57.3% (47.8–66.9%), when males of both strains were exposed to a group of two or 20 GA females, respectively. Because these confidence intervals include 50% (exactly equal mating competitiveness), mating competitiveness between OX4319L and GA are not significantly different. These results are similar to those seen in previous laboratory mating studies (Ant et al., 2012).

#### Intrinsic Population Growth

The intrinsic growth rate of a population, measured as the cumulative increase in the number of females from one generation to the next, was not affected by whether GA females mated with GA or OX4319L males (*P*_mate_ = 0.2263). The intrinsic growth rate (measured as the total number of larvae produced per generation) for GA females mated to OX4319L males was 920.9 and 1,031.5 for GA females mated to GA males. Although the cumulative number of larvae produced was not significantly different, the cumulative number of female progeny produced would be, because only the male larvae from GA females mated to OX4319L males would survive to adulthood.

#### Longevity

**Figure 2:**
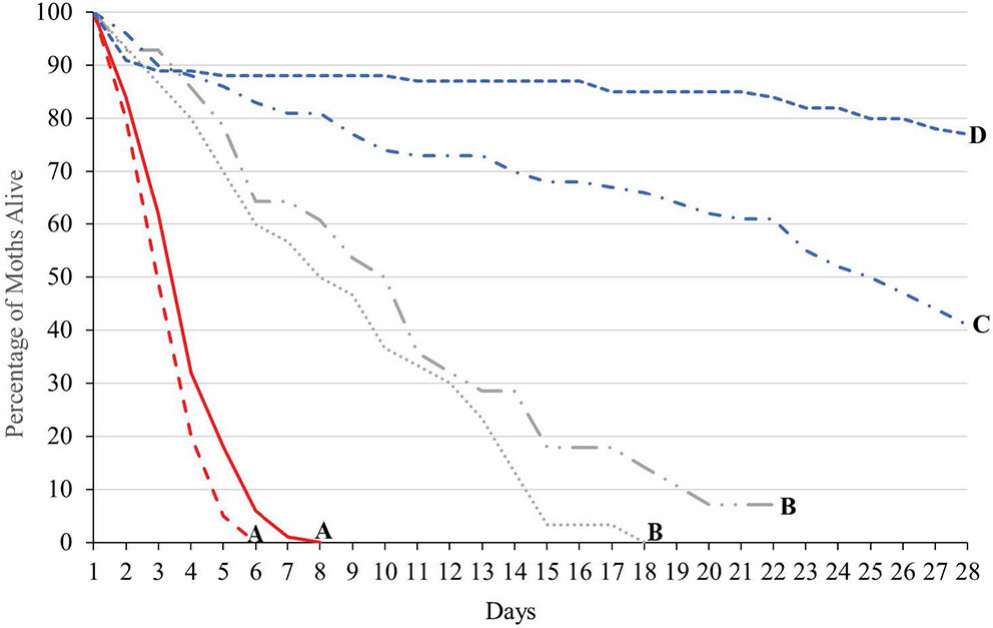
Longevity of 2 strains of *P. xylostella* under 3 different conditions in the laboratory: 100 males (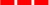, GA; 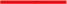, OX4319L) not provided 7.5% sugar water daily; 100 males (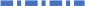, GA; 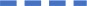, OX4319L) provided 7.5% sugar water daily; 28 GA females (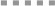, mated to GA males) and 30 GA females (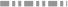, mated to OX4319L males) from intrinsic growth rate study (see text). Lines with the same letter are not significantly different as determined by Tukey's HSD test, HSD_0.05, 6_ = 2.87.

The longevity of each treatment group (males of either strain not provided with sugar water, males of either strain provided with sugar water and GA females mated to either strain and provided with cabbage juice to stimulate oviposition) was significantly different (Tukey HSD_0.05, 3_ = 2.35) (Figure 2). However, for males not provided sugar water, and for females mated to males from either strain, the longevity of both strains was not significantly different. When provided with sugar water daily, OX4319L males lived significantly longer than did GA males (Tukey HSD_0.05, 6_ = 2.87). However, for males not provided sugar water, and for females mated to males from either strain, the longevity of both strains was not significantly different. For GA females mated with OX4319L males or GA males, the median longevity was 8 and 10 days, respectively. The median longevity for GA males that were provided sugar water was 25 days. For the OX4319L males that were provided sugar water, 77% were still alive at 28 days. The median longevity of GA and OX4319L males without sugar water was 3 and 4 days, respectively.

Although the males supplied with sugar water lived longer than males without sugar water, this scenario is highly artificial and unlikely to be encountered under field conditions, where lifespan is anticipated to be significantly shorter. This increased longevity by OX4319L males is likely due to small differences in juvenile rearing conditions and/or adaptedness to laboratory rearing conditions: previous similar studies found the longevity of OX4319L males to be significantly lower than that of males from the same genetic background, reared under similar conditions (Jin et al., 2013).

#### Modeling Studies

**Figure 3:**
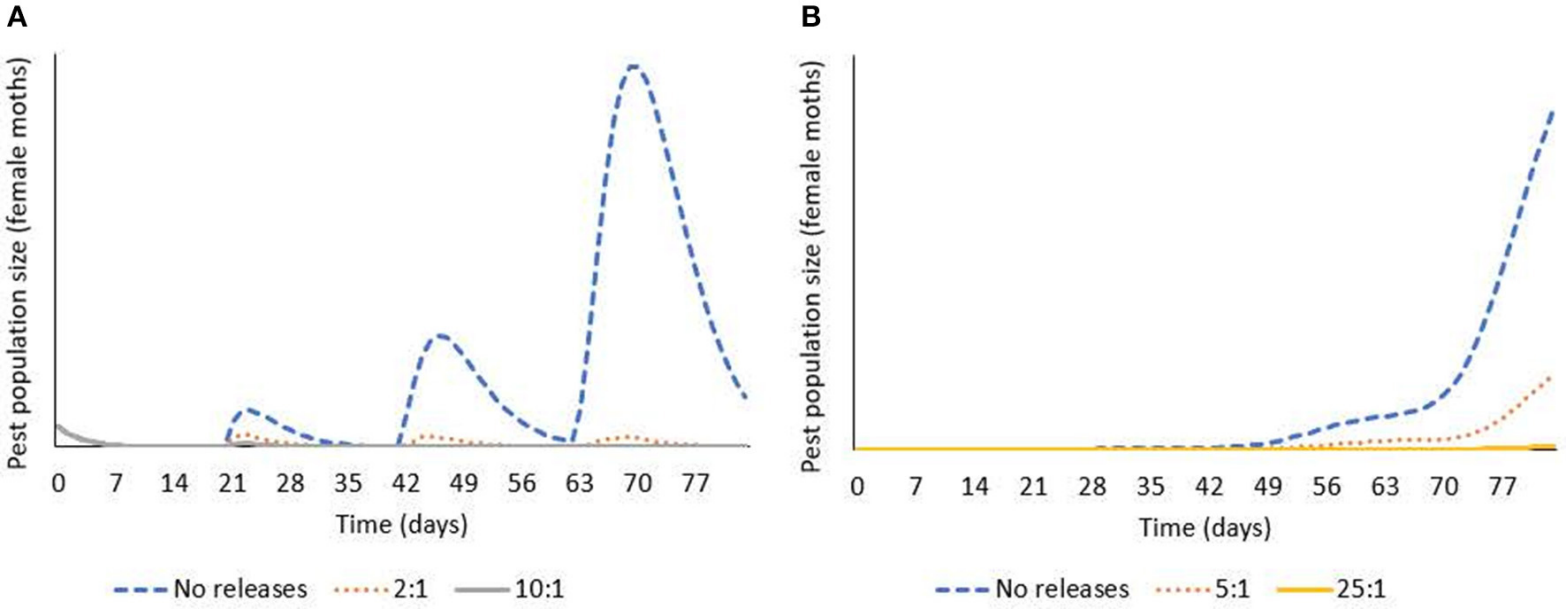
Deterministic modeling shows releases of OX4319L males suppress pest populations of *P. xylostella*, in which bi-weekly releases of OX4319L male moths are conducted from day 0. **(A)** Response of pest population growth after a single immigration event (on day 0) to releases of OX4319L males at different initial over-flooding rates (2:1 and 10:1), and no releases of OX4319L males. **(B)** Response of pest population growth to gradual immigration (days 1–21) to releases of OX4319L males at different initial over-flooding rates (5:1 and 25:1), and no releases of OX4319L males.

Integrating the results from lab and field studies, predictive deterministic modeling indicates that bi-weekly releases of OX4319L males will effectively suppress populations of pest *P. xylostella* in the field (Figure 3). In temperate regions of the USA, where cold winters prevent year-round presence of *P. xylostella*, the pest immigrates from southern states early in the growing season. In one iteration of the model, in which a single influx of *P. xylostella* occurs—for example, on contaminated seedlings (Shelton et al., 1996)—an initial over-flooding rate as low as 2:1 (calculated as ratio of OX4319L released relative to number of immigrating wild males, with bi-weekly releases thereafter) prevented expansion of the *P. xylostella* population. In a second iteration, in which immigration of wild *P. xylostella* occurs more gradually over 3 weeks, the effective over-flooding rate was higher, with 25:1 achieving significant suppression (calculated relative to weekly number of immigrating wild males).

## Discussion

These studies describe the first open-field release of any self-limiting insect in North America, and the first open-field release of a self-limiting agricultural pest in the world. Overall, these results are significant because they provide empirical evidence of how far these transgenic moths traveled and persisted under the field conditions encountered during our trial. These results are similar to results from other field releases of wild-type *P. xylostella* moths. These results provide evidence of the expected persistence and spatial limitations of OX4319L moths in the field under similar conditions. From a management perspective, the results suggest that released OX4319L moths will largely remain in the area of the field into which they are released.

Overall, based on the number of moths trapped, the behavior of male OX4319L moths in the field was very similar to that of a strain (GA) collected from Georgia, in the southern USA, where *P. xylostella* is a perennial pest (Philips et al., 2014). Most importantly, at any given release rate the predicted persistence in the field did not differ between strains (Table 1). Both strains showed similar movement patterns in the field, with 94.2% (GA) and 95.4% (OX4319L) of the recaptured moths occurring within 35 m of the release point during the 2 week release period (Table 5, Figure 1). Of the 10,000 OX4319L and 9,900 GA *P. xylostella* released during the entire study, <1% of either strain was recaptured at 95 m (Table 8), suggesting that the field size was appropriate for these mark-release-recapture studies with *P. xylostella*. As a requirement stipulated by the USDA Animal and Plant Health Inspection Service for this study, pheromone traps were also placed outside the field with four traps placed at cardinal points at 0.25, 0.5, 0.75, and 1 km beyond the field border. No OX4319L moths were detected on any of these traps. A single GA moth was found 0.25 km beyond the field, suggesting that this strain's dispersal was also limited.

The proportion of OX4319L recaptured was very similar to that recaptured for a wild-type strain (“Vero Beach,” the same genetic background as OX4319L) reported in a similar study by our program (Bolton et al., 2019), a study conducted prior to the field releases of OX4319L described here. Even though the present study deployed a higher number of pheromone-baited traps than in the previous study with the Vero Beach strain, it is worth noting that the transformed strain was recaptured in a distribution similar to its progenitor strain in these separate trials, again suggesting that adding the self-limiting trait did not affect its field behavior. The OX4319L males reared for this study exhibited similar eclosion and pupal rates and moths showed no obvious reduced activity in cages prior to release. Although a previous laboratory study found that the OX4319L transgene imposes a low fitness cost on the strain, our findings indicate that, as in a previous study (Somerville et al., 2019), in other experimental contexts this small fitness cost does not appear to significantly affect OX4319L male performance.

Additional confidence for our results with OX4319L is provided by a mark-release-recapture study of wild-type moths conducted in Australia (Mo et al., 2003). In that study the average distance traveled by *P. xylostella* before being recaptured on pheromone traps varied between 22 and 35 m over five releases. Although that study was conducted under different conditions—including trap design, crop and environmental conditions—the distance traveled by *P. xylostella* in that study is remarkably similar to the results from our study (Table 8).

Besides the distance traveled, it is also important to consider how long the moths persisted in the field. In the present study, both strains persisted in the field for a similar time when released at rates of 1,000 and 1,500 males per release and persisted significantly longer than those released at a rate of 2,500 males per release (Table 1). The reason for lower persistence at the higher rate remains unclear. The mean distances traveled by OX4319L and GA males were not significantly different at the 1,000 and 2,500 release rates but was different at the 1,500 release rate (Table 6). The reason for this difference also remains unclear. Most importantly, however, both strains were largely contained within the 2.83-ha field. In the context of evaluating the potential future field efficacy of OX4319L in a field release, our data suggest that OX4319L male dispersal was comparable with that of GA males.

Our field results indicate that, with 95% confidence, 75% of OX4319L males released at a rate of 1,500 could be expected to live between 3.5 and 5.4 days (Table 1) and 95% of these males could be expected to be detected within 25.8–34.9 m from the release point (Table 7). The mean distance traveled for OX4319L at this release rate was 39.3 m only 2 days after release (Table 6). For future suppression programs using OX4319L, these data suggest that releases, with either spatially continuous releases, or releases from discrete points 70 m apart, would provide appropriate coverage for every 0.25 ha of a given brassica field.

OX4319L and GA males had similar limited spatial dispersal and persistence under the conditions of this study. This is an important finding for implementation on farms. Control tactics, such as insecticide sprays, are usually deployed on a localized basis (e.g., a field). The site selected for the release was an isolated field surrounded by woods on three sides, including the side from which wind normally originates, and this may have helped to limit moth movement beyond the field border. Additionally, no storms with increased winds occurred during the field tests that might have increased dispersal. Although there is evidence of long-distance movement of *P. xylostella*, primarily in high altitude winds (Talekar and Shelton, 1993), studies have indicated that movement within a suitable and stable habitat is limited (Mo et al., 2003; Musser et al., 2005; Bolton et al., 2019).

Our laboratory studies indicate that OX4319L males are equally competitive as GA males in mating with GA females. Such competitiveness is in contrast to SIT programs in which released males are usually less competitive and therefore have to be released at higher rates (Rendón et al., 2004; Bakri et al., 2005). Furthermore, in the present study both strains had similar lifespans under similar conditions, except OX4319L isolated males lived significantly longer than GA males when the former are given sugar water daily. The longevity of males without sugar water in the laboratory (6–8 days) is similar to the maximum survival of 8.8 days predicted by the regression equation for field survival. This 8.8 day survival in the field is very close to the 8.1 day field survival obtained in an Austrailan study (Mo et al., 2003). Another finding in the present study is that mating with OX4319L males does not appear to have any effect on the fecundity of wild-type females. Collectively these results indicate that, aside from female mortality in the absence of tetracycline in larval feed, the life history of the OX4319L strain is representative of unmodified *P. xylostella* moths, with no observed significant performance constraints.

Population modeling, incorporating data from these field and laboratory studies, indicates that sustained releases of the OX4319L strain (released twice per week, at initial over-flooding rates of 2–25:1 OX4319L males for every wild diamondback male moth in the target population) will lead to significant population decline over >3 generations (Figure 3). The model suggests that this pest management strategy can be flexible and adapted to a variety of invasion scenarios and different infestation levels while remaining efficacious. It should also be noted that these overflooding rates are lower than is typical for the SIT: for example, pink bollworm, *Pectinophora gossypiella* (60:1) (Walters et al., 1998); codling moth, *Cydia pomonella* (40:1) (Proverbs et al., 1982); and painted apple moth, *Orgyia anartoides* (100:1) (Suckling et al., 2002; Wee et al., 2005). Notwithstanding that efficacy-related field studies will provide more robust estimates of effective over-flooding rates with OX4319L, self-limiting insects are anticipated to offer improved performance compared to radiation-sterilized insects.

In addition to the control achieved by releasing the OX4319L strain alone, it should be noted that this biological control method can be combined with other biopesticides to achieve more sustainable management of *P. xylostella* populations. For example, our modeling did not account for the complementarity expected between, for example, releases of self-limiting male *P. xylostella* and application of the biopesticide, Bt, which targets the larvae of Lepidoptera while leaving adults unaffected. In the context of an integrated pest management (IPM) program, required release rates of OX4319L males are likely to‘be lower than modeled here due to the additional pest suppression effect provided by other modes of action, as indicated by previous glasshouse studies (Harvey-Samuel et al., 2015). In addition, releases of OX4319L would provide a resistance management benefit, where the efficacy of insecticides, such as Bt, is threatened (Alphey et al., 2007; Harvey-Samuel et al., 2015).

These integrated field, laboratory and modeling studies suggest promise for application of OX4319L for crop protection programs against *P. xylostella*. Further field studies are recommended to demonstrate the potential for this self-limiting *P. xylostella* to provide pest suppression and resistance management benefits, as previously demonstrated in greenhouse studies (Harvey-Samuel et al., 2015).

To be sustainable, agriculture needs to adopt a broader IPM approach to reduce reliance on insecticides. These results suggest this self-limiting strain may provide an effective management tool by itself on Brassica crops and improve the efficacy of chemical or plant-based insecticidal methods through resistance dilution.

## Data Availability Statement

All data generated or analyzed during this study are included in this published article and accompanying files.

## Author Contributions

AS, SL, MB, and NM designed the study. AS, SL, AW, MB, HC, and LR performed the research. LJ, SL, and NM analyzed the data. NM constructed the model. AS, SL, LJ, and NM wrote the paper.

### Conflict of Interest

AW, MB, LR, and NM were employed by company Oxitex Ltd. The remaining authors declare that the research was conducted in the absence of any commercial or financial relationships that could be construed as a potential conflict of interest.
